# Differences in kinematic and match-play demands between elite winning and losing wheelchair padel players

**DOI:** 10.1371/journal.pone.0233475

**Published:** 2020-09-18

**Authors:** Daniel Navas, Santiago Veiga, Enrique Navarro, Jesús Ramón-Llín

**Affiliations:** 1 Faculty of Sport Sciences, Universidad Europea de Madrid, Madrid, Spain; 2 Health and Human Performance Department, Technical University of Madrid, Madrid, Spain; 3 Didactics of Musical, Visual and Body Expression Department, University of Valencia, Valencia, Spain; Toronto Rehabilitation Institute - UHN, CANADA

## Abstract

We aimed to compare the match-play and kinematic demands of the translation and rotation movements of elite wheelchair padel players as a function of match results. Twenty-two elite male players were video-analysed with a two-dimensional direct linear transformation (DLT) -corrected video system across seven matches of a professional tournament. Distance, turns, changes of direction, linear and angular speed, acceleration and the players’ heart rate (HR) were recorded. Losing couples in wheelchair padel covered greater distances than winners (*P <*0.001*; r =* 0.024) and did so at a higher speed (*P <*0.001; *r =* 0.06), while making greater efforts by accelerating (*P <*0.001; ∅ *= -*0.021), braking (*P <*0.001; ∅ *= -*0.014), and remaining less time stationary (*P <*0.001; ∅ *=* 0.059). In addition, losers performed more turns per rally (*P <*0.001; *r* = 0.04) at a faster speed, greater angular accelerations *(P <*0.001*; V =* 0.06) and greater average (*P* = 0.007; *d* = 0.91) and maximum (*P* = 0.20; *d* = 0.69) HR values. These data suggest that winner couples performed a better court positioning and employed a strategy to move the opponent during rallies in order to avoid them optimally reaching the ball.

## Introduction

Padel is a racket sport played in couples on a small-sized grass court (20 x 10 m), surrounded by glass and mesh walls. In recent years, it has become one of the sporting disciplines with a great increase in the number of practicants [1] and, consequently, a number of research studies have been conducted to analyse padel performance and match demands [1, 2]. Wheelchair padel is an adapted discipline from conventional padel which emerged in 2010 and where players are allowed to have a double bounce of the ball before returning it to their opponents (the same as in wheelchair tennis). This rule (double bounce) seems to be a relevant variable in relation to the players’ level and their performance, as wheelchair tennis players have been reported to make more errors when hitting the ball after zero bounces and more winners after two [3].

Previous research on padel performances have described the kinematic match demands of elite players, who spend between 60 and 90 minutes [4, 5] and cover around 2000 m per match [4, 6], with a majority of movements forward or sideward. Average players velocities are lower than 0.83 m/s for a half of match duration whereas they oscillate between 0.80 and 1.70 m/s for a third of matches. In relation to match-play demands, average heart rate (HR) values of elite padel players both in simulated or real matches range between 130–160 beats/min (bpm) [4, 6–9]. In wheelchair padel, there is only one precedent in literature [10] indicating that elite players covered 729.61 ± 369.90 m and turned 238.45 ± 129.45 times per match with effective playing times of around 29%. Players spend most of the match duration moving in low velocity zones (<0.50 m/s), with average match velocities of 0.53 ± 0.49 m/s. The match-play demands of this discipline have not been reported yet but elite players in other wheelchair racket disciplines obtained average HR values of between 120 and 140 bpm during matches [11–15]. It should be noted that, for the wheelchair disciplines, the calculation of the kinematic demands must include both linear and angular displacements, as both components are neccessary to describe the physical demands of players [16]. To date, however, few studies have examined the turns performed by wheelchair players during playing dynamics [17–20].

When match demands in racket sports have been examined according to the match results, winner couples in squash have been reported to cover shorter distances than the opponents [9]. On the other hand, winner players of tennis [21], padel [8] and wheelchair tennis [18, 22] disciplines have been reported to cover longer distances with faster average and maximum velocities. With regard to the match-play demands data, wheelchair tennis players who lost their matches obtained greater HR values than winners [15], although no other examples in the literature were found. Winner players could apply movement pressure on their opponents during rallies [23] as a tactical strategy in order to make losers cover longer distances. On the other hand, longer distances covered by winners could be associated with better defensive positioning and strategy as well as greater physical conditioning [21].

In order to obtain a better understanding of match-play and kinematic demands in the specific wheelchair padel discipline, the purpose of the present research was to compare the match-play and kinematic demands of the translation and rotation movements of elite wheelchair padel players as a function of the match result. We hypothesized that losing couples would obtain greater values in the match-play and kinematic match demands, in line with previous findings in racket disciplines.

## Materials and methods

### Participants

A total of 799 points of 126 games and 16 sets were analysed in seven official matches of the Professional World Chair Padel (WCP) circuit. Participants (N = 22) were male players whose characteristics (for winners or losers groups) are presented in Table 1. No differences were observed between winners or losers, except in the ranking variable (*P <*0.001). Approval for the study was gained from the Technical University of Madrid Research Ethics Committee and all participants provided their written informed consent to participate in the current investigation.

**Table 1:** Descriptive characteristics for wheelchair padel players.

Subject	Age (y)	Result	Weight (kg)	Nature of disability	Years since injury	Years practicing padel	Point classification	Rank
1	41	L	93	Thoracic SCI (T4)	10	2	1	Top 40
2	37	L	78	Thoracic SCI (T4)	13	2	1	Top 50
3	31	L	62	Thoracic SCI (T6)	3	2	1	Top 30
4	43	L	74	Thoracic SCI (T4)	12	4	1	Top 40
5	27	L	80	Thoracic SCI (T11)	5	3	2	Top 30
6	44	L	70	Thoracic SCI (T5)	24	14	2	Top 30
7	44	L	76	Thoracic SCI (T11)	24	12	2	Top 20
8	35	L	89	Spina bifida	6	4	2	Top 20
9	33	L	63	Sacral agenesis	33	10	2	Top 30
10	53	L	72	Poliomyelitis	20	15	3	Top 50
11	51	L	90	Cerebral palsy	11	5	3	Top 40
12	32	L	86	Muscular atrophy	13	7	4	Top 20
Mean	39.25		77.25		14.50	6.67	2.00	
SD	8.10		10.26		9.00	4.85	0.95	
13	42	W	78	Thoracic SCI (T6)	10	6	1	Top 20
14	39	W	60	Thoracic SCI (T5)	21	8	1	Top 10
15	39	W	65	Thoracic SCI (T9)	17	6	2	Top 10
16	43	W	75	Thoracic SCI (T12)	9	8	3	Top 10
17	35	W	60	Amputation	37	17	2	Top 10
18	42	W	81	Hip disarticulation	4	2	3	Top 10
19	60	W	65	Poliomyelitis	3	2	2	Top 10
20	44	W	75	Amputation	3	2	4	Top 10
21	31	W	75	Lumbar SCI (L2)	11	3	4	Top 30
22	33	W	91	Amputation	8	4	4	Top 10
Mean	40.8		72.50		12.30	5.80	2.60	
SD	8.05		9.94		10.47	4.59	1.17	

Abbreviations: W = winner; L = loser.

### Data acquisition and processing

The matches were filmed using two digital video cameras JVC® GZ-MG (Japan Victor Company, Japan) recording at 25 Hz each, located at the top of each front wall court (4 metres above and right in the middle of the court) and with the optical axis pointed at the opposite half of the court [10]. Match footage was analysed with a two-dimensional DLT-corrected [24] video system that allowed the calculation of the players’ real coordinates over the court surface plane (in metres) from the two-dimensional screen coordinates (in pixels). Using the software Photo23D [25], an experimental observer manually digitized (at a sampling frequency of five frames per second) the point of contact of the two wheels of each player’s wheelchair during the match duration. This method has been extensively described in previous performance analyses of different able-bodied disciplines, such as football [26], track and field [27] and swimming [28]. Once the two-dimensional coordinates of the players’ trajectories were obtained, they were smoothed using quintic splines functions with the cross-generalized validation procedure as a method for evaluating the adjusting factor [29].

Before the beginning of matches, five control points uniformly distributed along each playing court and represented by field markers were filmed and employed for calibration purposes. The validity of the measurement system was checked by calculating the root mean square error (RMSE) when reconstructing two control points not used for calibration purposes. Position RMSE at 30 different dispositions was 0.03 ± 0.04 m which, at a frame rate of 5 Hz, corresponded to a velocity RMSE of 0.15 m/s and an acceleration RMSE of 0.75 m/s^2^. In order to determinate the reliability of the method, the experiment observer repeated the digitization of the same rally sequence30 times. The intra-reliability [30] tests did not reveal any significant differences (P <0.05) between observations, with the coefficient of variation in the measurements consistently less than 1%.

Match analysis was limited to the effective playing time. This was defined as the playing time of each rally from a player hitting the ball at serve until the point has ended (according to the WCP regulations). The kinematic variables obtained from the video analysis were: 1) total distance (m) calculated as the linear trajectory of the middle point between the two contact points of the wheelchair with the court; 2) chair turns (360°) calculated as the angular displacement of the planar vector joining the same two aforementioned contact points; and 3) mean and peak rally speed (m/s) from the first derivative of the position data of players. Chair turns were also analysed according to the positive or negative angular displacement of the wheelchair during rallies, expressed as change of direction. For the calculation of speed zones, incremental ranges of 0.50 m/s were used in the same way as proposed in Sindall et al. [31], whereas acceleration profiles were calculated from increasing or decreasing instantaneous velocities data during rallies.

For the physical demands variables, the HR (bpm) of 22 players (11 winners and 11 losers) was recorded in 5-second intervals via short-range radio telemetry using a Polar RS 400 (Polar, Kempele, Finland) and was exported with the Polar Pro Trainer 5 program [32] to spreadsheets (Microsoft Excel 2013, Redmond, USA). Maximum, minimum and average HR values were calculated for each individual game and used to calculate average values for each set or match play. The standard formula for the calculation of age-predicted maximum HR (220—age) was applied to determine the percentage of age-predicted maximum HR, as previously done in wheelchair tennis players [13, 15]. HR zones were established according to previous studies [33] as low (<75% of max-HR), moderate (75–85% of max-HR), high (85–95% of max-HR) and maximum (>95% of max-HR).

### Statistical analyses

Statistical analysis was performed with SPSS 24.0 (Chicago, Illiois, USA). Kinematic and match-play parameters were measured individually and then averaged from each couple during the match. First, normality (Kolgomorov–Smirnov) and homoscedasticity (Levene) tests were performed to determine whether the analysed variables had a parametric distribution. For the linear and angular velocities, distances in metres, chair turns and changes of direction (non-parametric distribution), Wilcoxon tests were performed to compare winning versus losing couples. Effect sizes were calculated from r values considering <0.10, 0.10–0.30, 0.30–0.50 and >0.50 thresholds for trivial, small, moderate and high magnitudes [34]. For the linear and angular acceleration profiles as well as the HR intensity profile, chi-square tests were performed with post hoc tests adjusting the value of significance according to Bonferroni. The effect size on these variables was calculated from Crammer’s V and phi for post hoc tests, where 0.1 represented a small effect, 0.3 medium and 0.5 large [35]. Finally, for the mean HR, maximum HR and max-to-mean ratio (parametric distribution), T tests on related samples were performed and effect sizes were computed as a function of Cohen’s d [36] (being <0.20, 0.20–0.50, 0.50–0.8 and >0.80 the thresholds for trivial, small, moderate and high differences). The significance values were established from p <0.05.

## Results

**Fig 1:**
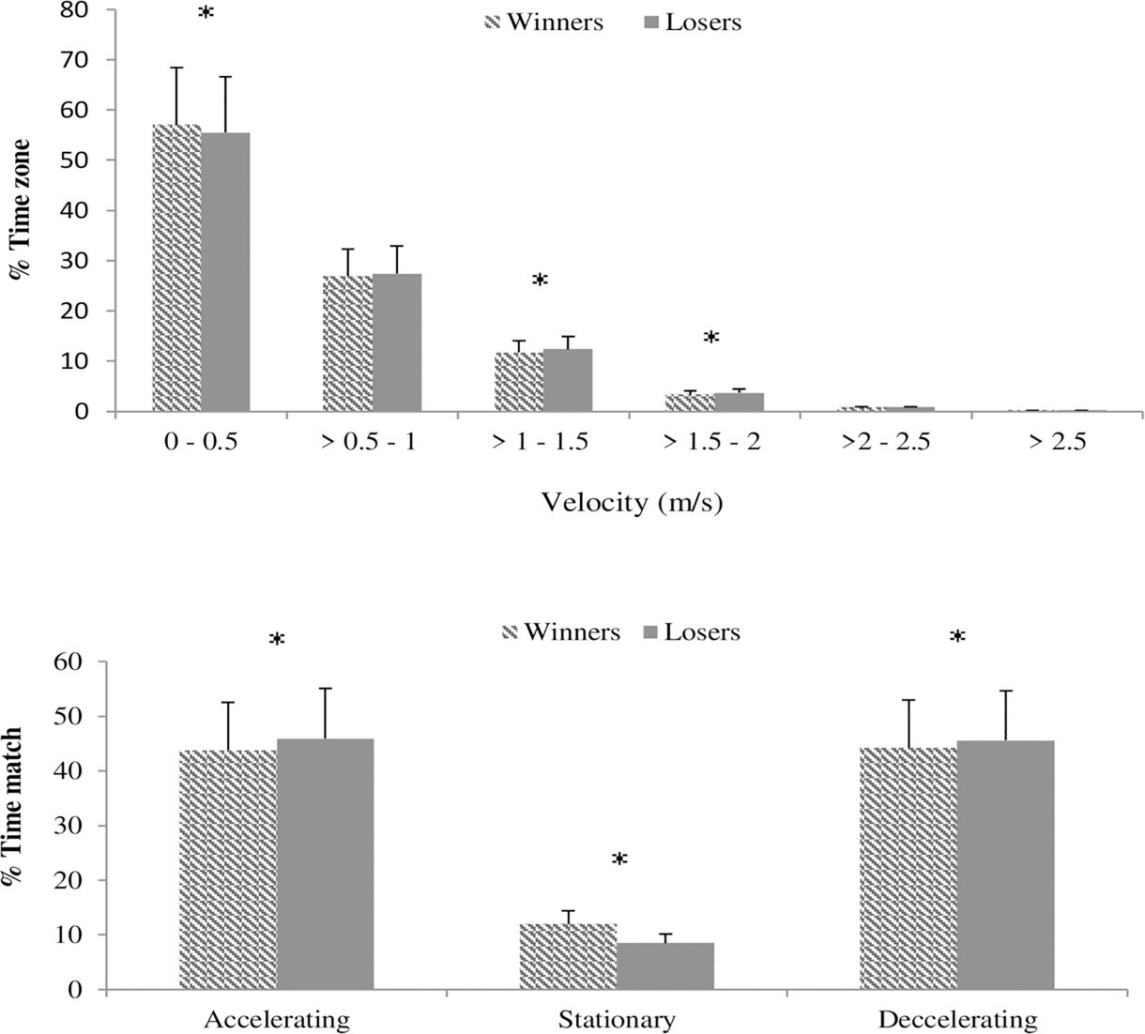
a (Above): Velocity profiles of elite wheelchair padel players according to the match result. b. (Below): Time spent on the increasing-decreasing velocity or standing stationary categories for elite wheelchair padel players according to the match result (%). * P < 0.05.

Distances covered during the rallies of the losing couples during elite wheelchair padel matches were greater (6.55 ± 6.54 vs 6.24 ± 6.34; Z = -3.92; *P <*0.001; *r* = 0.024*)* than the winners, with no statistical differences during games or sets despite the same tendency (Table 2). In the same vein, mean speed during matches was faster (0.57 ± 0.51 m/s vs 0.51 ± 0.47 m/s; *Z* = -5.10; *P <*0.001*; r =* 0.06*)* for the losing couples than for the winning ones (Table 2). Velocity profiles (Fig 1A), indicated that both groups (losers and winners) spent most of the match time in low-velocity zones (<0.50 m/s) but there were statistical effects due to performance (*χ^2^*_*5*_ *=* 37.97*; P <*0.001, *V =* 0.16). Post hoc tests indicated that losing couples spent less time moving below 0.5 m/s (*χ^2^*_*1*_ *=* 27.05; *P <*0.001; ∅ *=* 0.013).) and longer time between 1.00 and 1.50 m/s (*χ^2^*_*1*_ *=* 16.0*; P <*0.001; ∅ *=* 0.010) and 2.00 and 2.50 m/s (*χ^2^*_*1*_ *=* 10.80*; P <*0.001; ∅ *=* 0.008) than winners. For the changes in velocity (*χ^2^*_*2*_ *=* 547*; P <*0.001; *V =* 0.059*)*, losing couples spent more time during matches accelerating (45.90% losers vs 43.80% winners; *χ^2^*_*1*_ *=* 72.9*; P <*0.001; ∅ *= -*0.021) and decelerating (45.60% losers vs 44.20% winners; *χ^2^*_*1*_ *=* 32.5*; P <*0.001; ∅ *= -*0.014) and they also spent less time standing stationary than winners (8.50% vs 12.30%; *χ^2^*_*1*_ *=* 54.0; *P <*0.001; ∅ *=* 0.059) (Fig 1B).

**Table 2:** Descriptive statistic for distance and velocity for wheelchair padel players.

Variable	Group	Mean	SD	p	ES
**DR (m)**	Losers	6.5	6.5	<0.001	r = 0.024
	Winners	6.2	6.3		
**DG (m)**	Losers	41.6	32.4	0.078	r = 0.073
	Winners	39.5	24.5		
**DS (m)**	Losers	327.3	159.9	0.186	d = 0.112
	Winners	311.1	127.3		
**Speed (m/s)**	Losers	0.57	0.51	<0.001	r = 0.06
	Winners	0.51	0.47		

Abbreviation: DR—distance per rally; DG—distance per game; DS—distance per set; ES: effect size.

**Fig 2:**
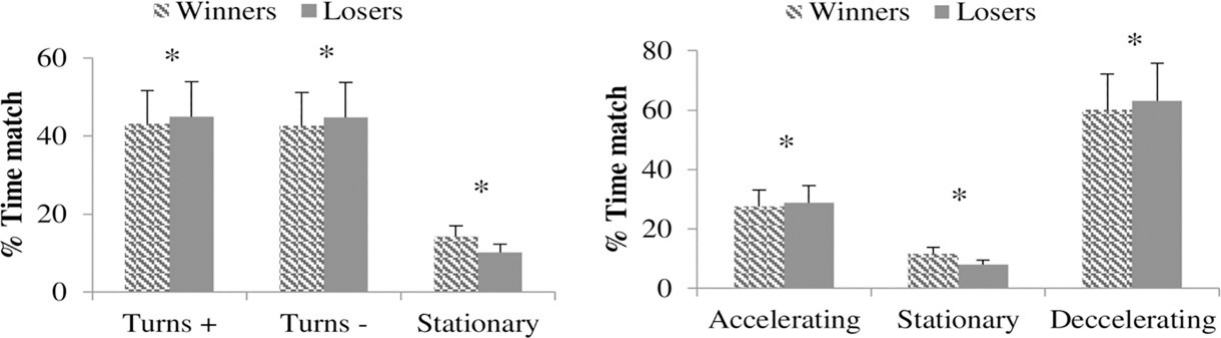
a (Left): Time spent on the positive-negative turning or standing stationary categories by elite wheelchair padel players (%). b. (Right): Time spent on the increasing-decreasing angular velocity or standing stationary categories for elite wheelchair according to the match result (%). * P < 0.05.

For the angular motion, losing couples performed a statistically greater number of turns than winners during rallies (*Z* = -4.87; *P <*0.001; *r* = 0.04), games (*Z* = -2.26; *P <*0.024; *r* = 0.06) and sets (*Z* = -1.52; *P <*0.042; *r* = 0.09) at the same time as performing a greater number of changes of direction (*Z* = -4.90; *P <*0.001; *V =* 0.06 for the counter-clockwise) and (*Z* = -5.40; *P <*0.001; *V =* 0.07 for the clockwise turns) during wheelchair padel matches. Among all turns, losing couples performed 14.71 ± 13.61 anticlockwise and 14.83 ± 13.62 clockwise turns during matches, whereas winners performed 13.92 ± 13.02 and 13.94 ± 13.10, respectively. Angular velocities during matches were statistically faster (5.48 ± 6.50°/s Vs. 4.77 ± 5.91°/s; *Z* = -4.10; *P <*0.001*; r =* 0.06) for the losing couples compared to winners (5.48 ± 6.50°/s losers and 4.77 ± 5.91°/s winners) and analysis of time spent on the positive–negative turning and time standing stationary (Fig 2A) showed significant differences (*χ^2^*_*2*_ *=* 604.19; *P <*0.001; *V =* 0.062), where losers spent a significantly longer percentage of time turning for clockwise (positive) (45.0% losers vs 43.1% winners; *χ^2^*_*1*_ *=* 58.2*; P <*0.001; ∅ *=* 0.019), anticlockwise (negative) (44.8% losers Vs 42.7% winners; *χ^2^*_*1*_ *=* 73.4*; P <*0.001; ∅ *= -*0.022) and standing stationary (10.2% losers Vs 14.2% winners; *χ^2^*_*1*_ *=* 603.0*; P <*0.001; ∅ *=* 0.062). For the changes in angular velocity, losing couples spent a longer proportion of matches (*χ^2^*_*2*_ *=* 566.70*; P <*0.001*; V =* 0.60) accelerating (28.80% vs 27.60%; *χ^2^*_*1*_ *=* 31.6*; P <*0.001; ∅ *= -*0.014) and decelerating (63.10% vs 60.80%; *χ^2^*_*1*_ *=* 87.9*; P <*0.001; ∅ *= -*0.024) but shorter standing stationary (*χ^2^*_*1*_ *=* 566.2*; P <*0.001; ∅ *=* 0.060) than winners (Fig 2B).

**Fig 3:**
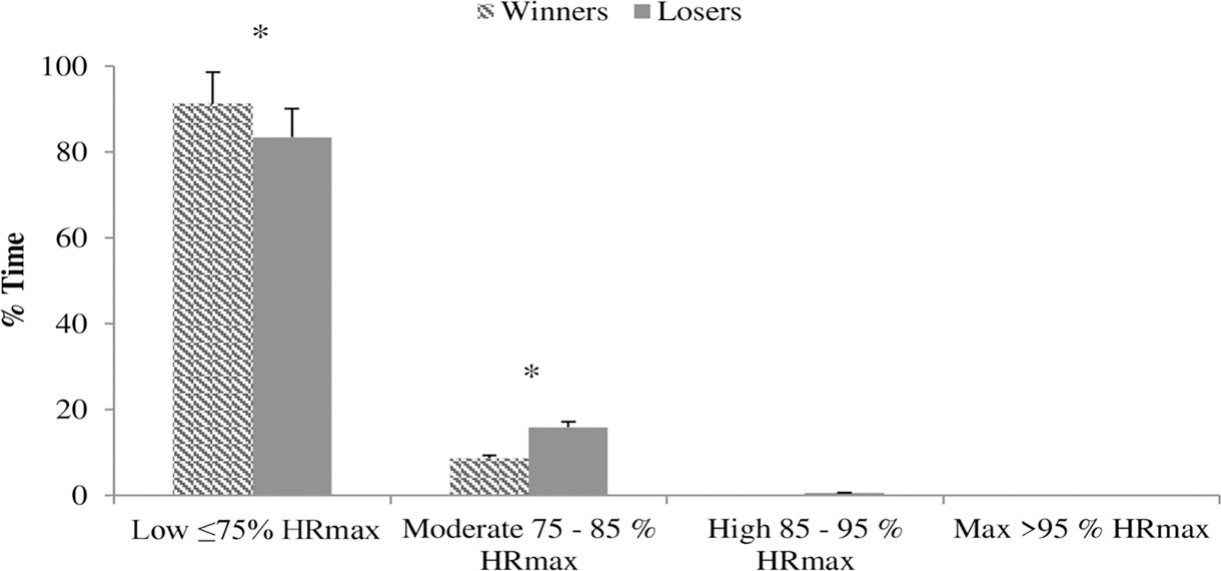
Time spent on the HR intervals by elite wheelchair padel players according to the match result (%). * P < 0.05.

The mean HR during elite wheelchair padel matches of the losing couples was statistically greater than that of the winners (123.05 ± 12.92 vs 113.83 ± 12.58 bpm; *P* = 0.007, *d* = 0.91), observing differences in the maximun HR values (181.01 ± 11.02 vs 174.71 ± 10.72 bpm; *P* = 0.20, *d* = 0.69) and the mean to max HR ratio (67.98 ± 1.81 vs 65.16 ± 5.22%; *P* = 0.92, *d* = 0.05). Theoretical HR-max (220-age) for the loser couples was 182 ± 6.61 and for the winners 175 ± 10.20 bpm, thus there were no statistical differences (T_20 =_ -0.448; *P =* 0.659). Time spent (Fig 3) in high-intensity effort zones (in relative terms to max-HR) reported significant differences (*χ^2^*_*2*_ *=* 136.60*; P <*0.001; *V* = 0.12*)*. Time spent in low-intensity effort zones was less for the loser couples (83.48% vs 91.31%; *χ^2^*_*1*_ *=* 124.5*; P <*0.001; ∅ *=* 0.118) than for the winners, but the opposite was found for the moderate-intensity effort zone (15.95% vs 8.69%; *χ^2^*_*1*_ *=* 109.4*; P <*0.001; ∅ *=* 0.111).

## Discussion

Although kinematic and physical match demands can provide great insight on improving players performance [14], there are no scientific evidences in the discipline of wheelchair padel. Therefore, the aim of the present research was to compare the match-play and kinematic demands of the translation and rotation movements of elite wheelchair padel players as a function of the match result. Results indicated that losing couples obtained greater values on the kinematic performance indicators, with a higher physical load than winners.

Loser wheelchair padel players covered approximately 0.5 m longer distances per rally than winners, probably related to the ability of winners to tactically move the opponent during the rally, as previously observed in the squash [9, 23] and tennis disciplines. These differences were not observed in distances covered by game or set, as matches could be composed of a different number of games or sets depending on the result [9]. In wheelchair tennis, on the other hand, Sindall et al. [15], Filipcic and Filipcic [22] and Dinkelberg [18], observed that losers covered less distance than winners. Therefore, as previously stated in conventional tennis [37], playing couples in racket disciplines could perform different playing strategies depending on the opponent. Loser wheelchair padel players also exhibited faster average velocities (around 0.06 m/s) during matches than winners and they employed a greater proportion of match time playing at velocities of 1.0–1.5 m/s (and a lower proportion below 0.5 m/s). These data indicate a greater playing intensity of losers versus winner couples and reinforces the notion that winners employed a successful strategy consisting of moving the opponent, although previous data on wheelchair tennis [22] did not find velocity differences between the players’ match results (0.98 vs 0.87 m/s).

As well as linear kinematics, loser wheelchair padel players also performed a greater number of turns (≈1 more complete turn) than winner couples during matches. In other disciplines where wheelchair players directly interact during matches (i.e. wheelchair basketball or rugby), it has been observed that higher performance players exhibit better movement abilities and they perform more turns during rallies [17, 19]. Nevertheless, these are disciplines where the abilities of players to position themselves in the same way as the opponent or to move away from him/her (and therefore to exhibit great wheelchair movement abilities) are of critical importance. Compared to wheelchair tennis, players in the present research performed more turns per rally than data from one generic match of elite players reported by Dinkelberg [18]. This could be explained by the smaller field of play in padel compared to tennis [38] or the longer duration of rallies in wheelchair padel [10], although the different methodological definition of wheelchair turns in both studies could probably explain most of the differences. In relation to the angular velocities, loser couples in the present study also performed faster average velocities per rally (almost 1°/s) and they spent a greater proportion of the match turning than winners. Previously, De Witte et al. [17] had reported that wheelchair basketball winners spent more time without turning and averaged higher rotation speeds than loser players. However, to the best of our knowledge, no previous studies have presented the angular velocity values during wheelchair matches so no possible comparison with our data was made.

In line with greater kinematic demands of losing couples during elite wheelchair padel matches, the HR values of the losing players (123.05 ± 12.92 bpm, 67.98% of HR-max) were greater than those of the winners (113.83 ± 12.58 bpm, 65.16% of HR-max). Average HR values are a valid indicator of the accumulated physiological stress associated with the bouts of play [15] so probably loser players had a greater physical load during matches than winners. According to previous research in wheelchair tennis, where lower-standard players also exhibited greater HR values [15], an elevated playing intensity is likely to be caused by a combination of an opponent’s actions and poor court positioning strategies. However, as a lower submaximal HR response is associated with a higher level of aerobic fitness, these data may also indicate that the level of aerobic conditioning is a factor in determining match-play outcomes in lower-standard players [15].

Obtained average values of HR in the present research were lower than previously reported in conventional padel (between 130 and 160 bpm) players during competitive matches [6, 8]. although conventional and wheelchair padel are played with the same rest time between rallies, the HR values were lower in the wheelchair modality probably due to the use of upper limbs for locomotion and the lower velocity of play. This was also reported when comparing conventional and wheelchair tennis [14]. When comparing average HR values in the present research with those of wheelchair tennis players, values were higher in tennis (120–140 bpm) [11–14] probably due to the different court dimensions. However, it should be noted that HR analyses in wheelchair tennis matches correspond to individual (not doubles) matches, where both the HR and oxygen uptake tend to be higher [39]. Of course, the match length or other variables like the court surface could also influence the HR values and consequently the physiological match demands [40].

Beyond average HR values, the compilation of relative HR values compared to the theoretical maximum could provide even further understanding of the game intensity [12]. In the present research, both losing and winning couples spent most of the match time in low-intensity playing zones (83.48% and 91.31%, respectively) whereas the proportion of time in moderate-intensity (15.95% and 8.69%, respectively) and even in high-intensity zones was much lower. These values show how winning couples spent a greater match proportion playing at low intensity, which could be related to the strategy of moving the opponent during the rallies (in line with greater kinematic demands of loser couples). Compared to other wheelchair disciplines, data from basketball matches showed that players spent the longest time in zones IV (80–89%) and V (90–100%) of HR-max [41]. The fact that basketball matches present a higher work-to-rest ratio with actual playing time accounting for 50% of total match time (excluding substitution time) compared to the 28.72% of wheelchair padel [10] could explain differences. In the case of wheelchair tennis, players spent the longest time in zone V (57.90%) (> lactate turn point) for 15–20% of match time [13], although data were compiled from individual and not couples matches (as in the present research). This could lead to a greater playing intensity of tennis compared to wheelchair padel players.

## Limitations of the study

Results in the present research represent the first kinematic and match-play demands information available during elite wheelchair padel matches, in relation to the match result. Although the application of results could be high, data should be considered with caution because of various limitations: As might be expected, one of the major difficulties facing research in wheelchair sports is the small population available as well as the variation of injuries within population groups [12]. This resulted in a large dispersion of data and in trivial or small effect sizes for the majority of variables. Also, no previous stress tests to determine physiological parameters, such as maximum HR and anaerobic threshold of the players, were performed. This led to the use of 220-age formula to determine the HR zones, which undoubtedly could represent an important limitation. Future research examining the potential influence of various independent disability groups on activity profiles is advised to further understand wheelchair padel performance. Also, given that the chair configurations in the present research were not manipulated by the investigators for obvious reasons, it is important to note that there may have been differences in the rolling resistances experienced by individuals with their choice of tyre type and pressure [42], and this may have influenced energy expenditure or HR [15]. Finally, kinematic and match-play demands in the present research should be complemented by further information about the players during the game as well as the precision of the ball strokes.

## Conclusions

Losing couples of wheelchair padel playing in elite matches obtained greater values on the linear and angular kinematic performance indicators in all registration units (distance covered per rally, faster speeds or greater number of turns, and changes of direction) with greater values of average HR during matches as well as a greater proportion of time playing in higher-intensity effort zones. The magnitude of differences between couples was small for the kinematic parameters, although high or moderate differences were observed in relation to heart rate values. These data suggest that winner couples performed better court positioning and employed a strategy to move the opponent during rallies in order to prevent them from optimally reaching the ball. Results were partly different to other wheelchair disciplines in line with padel-specific playing dynamics and, therefore, allow both players and coaches to be aware of the activity profile of this growing discipline.

## Supporting information

S1 File Kinematic data. (XLSX)
